# Unlocking the synergistic potential of HAT activation and HDAC inhibition for rescuing memory deficits in Alzheimer’s Disease

**DOI:** 10.1002/alz.087929

**Published:** 2025-01-09

**Authors:** Elisa Calcagno, Elisa Zuccarello, Anna Staniszewski, Jonhatan van de Loo, Hong Zhang, Erica Acquarone, David K. Stack, Florence Haut, Shi‐Xian Deng, Donald W. Landry, Jole Fiorito, Ottavio Arancio

**Affiliations:** ^1^ Columbia University, New York, NY USA; ^2^ New York Institute of Technology, New York, NY USA

## Abstract

**Background:**

Memory is influenced by epigenetic mechanisms that regulate gene expression. Histone acetyltransferases (HATs), and histone deacetylases (HDACs), are two competitive enzymes regulating histone acetylation. Histone acetylation is reduced in Alzheimer’s disease (AD) brains, and evidence has shown a synergistic regulation of HDACs and HATs activities. Specific HATs such as CREB binding protein (CBP), p300, and PCAF positively influence memory, while HDAC2 and HDAC3 negatively impact it. Maintaining the balance between HAT and HDAC levels and activity is crucial for regulating gene expression profiles essential to memory processes. Consequently, increasing histone acetylation in neurons, while maintaining the balance between HAT and HDAC enzymes, offers the potential to attenuate AD progression by acting at the downstream level of Aβ and tau elevation.

**Method:**

Hippocampal slices from C57Bl6 mice were exposed to Ab oligomers (oAβ) plus the HAT activator YF2 and/or the HDAC inhibitor TSA in electrophysiological experiments assessing long‐term potentiation (LTP), a cellular surrogate of memory formation. The APP/PS1 mouse model of Aβ elevation and the hTau/Mapt‐KO mouse model of tau elevation were used in electrophysiological, behavioral and biochemical experiments aimed at investigating LTP and memory.

**Result:**

Following establishment of subthreshold doses (i.e. doses immediately below the concentration that has a minimal beneficial effect on LTP) for YF2 and TSA alone against Ab‐oligomer induced LTP defect, we found that pairing subthreshold doses of the two compounds rescued the oAβ‐induced LTP impairment. The beneficial effect of the combination was also present when subthreshold doses of YF2 and TSA were administered to 3‐month old APP/PS1 mice and 12 month old hTau/Mapt‐KO mice. Notably, chronic administration of YF2 (0.5 mg/kg) and TSA (0.5 mg/Kg) for 1 month in APP/PS1 mice and 3 months in hTau/Mapt‐KO animals rescued the defects in associative and spatial memory, as well as LTP.

**Conclusion:**

In summary, the synergistic effect observed with the combination of YF2 and TSA not only maintains the balance of HAT/HDAC activity but also enables the use of lower concentrations for both drugs, overcoming the specificity challenges associated with HDAC inhibitors. This compelling combination emerges as a highly promising therapeutic strategy against AD.

